# Test-retest reliability of the KINARM end-point robot for assessment of sensory, motor and neurocognitive function in young adult athletes

**DOI:** 10.1371/journal.pone.0196205

**Published:** 2018-04-24

**Authors:** Cameron S. Mang, Tara A. Whitten, Madeline S. Cosh, Stephen H. Scott, J. Preston Wiley, Chantel T. Debert, Sean P. Dukelow, Brian W. Benson

**Affiliations:** 1 Department of Clinical Neurosciences, Cumming School of Medicine, University of Calgary, Calgary, Alberta, Canada; 2 Hotchkiss Brain Institute, Calgary, Alberta, Canada; 3 WinSport Medicine Clinic, Winter Sport Institute, Calgary, Alberta, Canada; 4 Department of Biomedical and Molecular Sciences, Queen’s University, Kingston, Ontario, Canada; 5 Faculty of Kinesiology, Sport Medicine Centre, University of Calgary, Calgary, Alberta, Canada; 6 Canadian Sport Institute Calgary, Calgary, Alberta, Canada; Roskamp Institute, UNITED STATES

## Abstract

**Background:**

Current assessment tools for sport-related concussion are limited by a reliance on subjective interpretation and patient symptom reporting. Robotic assessments may provide more objective and precise measures of neurological function than traditional clinical tests.

**Objective:**

To determine the reliability of assessments of sensory, motor and cognitive function conducted with the KINARM end-point robotic device in young adult elite athletes.

**Methods:**

Sixty-four randomly selected healthy, young adult elite athletes participated. Twenty-five individuals (25 M, mean age±SD, 20.2±2.1 years) participated in a *within-season* study, where three assessments were conducted within a single season (assessments labeled by session: S1, S2, S3). An additional 39 individuals (28M; 22.8±6.0 years) participated in a *year-to-year* study, where annual pre-season assessments were conducted for three consecutive seasons (assessments labeled by year: Y1, Y2, Y3). Forty-four parameters from five robotic tasks (Visually Guided Reaching, Position Matching, Object Hit, Object Hit and Avoid, and Trail Making B) and overall Task Scores describing performance on each task were quantified.

**Results:**

Test-retest reliability was determined by intra-class correlation coefficients (ICCs) between the first and second, and second and third assessments. In the *within-season* study, ICCs were ≥0.50 for 68% of parameters between S1 and S2, 80% of parameters between S2 and S3, and for three of the five Task Scores both between S1 and S2, and S2 and S3. In the *year-to-year* study, ICCs were ≥0.50 for 64% of parameters between Y1 and Y2, 82% of parameters between Y2 and Y3, and for four of the five Task Scores both between Y1 and Y2, and Y2 and Y3.

**Conclusions:**

Overall, the results suggest moderate-to-good test-retest reliability for the majority of parameters measured by the KINARM robot in healthy young adult elite athletes. Future work will consider the potential use of this information for clinical assessment of concussion-related neurological deficits.

## Introduction

Accurate clinical assessment and appropriate management of sport-related concussion (SRC) remains a challenge in sport medicine [1,2]. Importantly, subtle deficits, if missed, may have significant consequences in a sport environment. A key source of difficulty for the field relates to the wide range of symptoms, which cross multiple neurological domains, and variable symptom severity that can be experienced post-SRC [2–4]. Adding to the challenge of assessing such a diffuse injury is that traditionally many clinical assessments of neurologic dysfunction rely on patient symptom reporting and subjective interpretation that may be influenced by examiners’ disciplines and prior clinical experience [5–7].

Developing assessments with good test-retest reliability poses another challenge to the field that is critical to overcome if such assessments are to be used to establish baseline levels of function and/or track individuals’ recovery post-SRC. For example, a large study of the Immediate Post-concussion Assessment Tool and Cognitive Test (ImPACT), one of the most commonly used SRC assessment tools in clinical and research settings [8], recently reported lower test-retest reliability than was previously thought (intraclass correlation coefficients [ICCs] for the four composite scores ranging from 0.36 to 0.90 across 1-, 2- and 3-year time intervals) [9]. Thus, there remains a need to develop valid clinical tools that are able to objectively, efficiently and reliably assess functional impairment across multiple domains.

Robotic technology offers a promising avenue for development of improved assessments of neurologic deficits post-SRC. Robotic tools can be designed to provide rapid and comprehensive assessments of neurologic function with a level of precision and accuracy not found in standard clinical assessments [5,10]. The Kinesiological Instrument for Normal and Altered Reaching Movements (KINARM, BKIN Technologies Ltd, Kingston, Canada) is a robotic device on which a set of standard assessments of sensory, motor and neurocognitive function have been developed [11–13]. Subsets of the KINARM Standard Tests (KST^™^) have been found to be reliable for neurologic assessment in adults with acute stroke [14–17], and sufficiently sensitive to detect impairments related to stroke [14–18] and moderate/severe brain injury [19]. Recently, a subset of the KINARM Standard Tests were also found to have generally moderate-to-good test-retest reliability in healthy pediatric ice hockey players when tested twice in a single day and once a week later [12]. The same KINARM Standard Tests were utilized in a study of mild traumatic brain injury (mTBI) presenting to the emergency department [20]. In this study, acute performance (<24 hrs post-injury) on KINARM assessments was predictive of persistent symptoms three weeks later [20], suggesting potential clinical utility of the KINARM in the mTBI population, a group that likely overlaps to a considerable degree with individuals with acute SRC. Nevertheless, the test-retest reliability of these KINARM assessments specifically in young adult athletes, and at long test intervals is yet to be determined.

The primary objective of the current study was to determine test-retest reliability of KINARM robotic assessments of sensory, motor and neurocognitive function in healthy, non-concussed, young adult elite athletes. As a SRC could occur with variable timeframes relative to a healthy baseline assessment, we evaluated test-retest reliability of three assessments conducted within a single season and across three consecutive seasons in two separate groups of athletes. Determining test-retest reliability of KINARM assessments in athletic populations is a crucial first step towards future application of robotics to this field.

## Materials and methods

### Participants

Participants were recruited from a larger sample participating in an ongoing prospective longitudinal study of SRC. For the prospective study, participants are recruited from varsity sports teams (football, hockey, wrestling, basketball, rugby, volleyball), junior hockey teams, national sports teams (alpine skiing, freestyle skiing, Nordic skiing, luge, skeleton, bobsled, speed skating, track and field, waterpolo, wrestling), a youth sport school, and national youth developmental programs for the sports listed above. Inclusion criteria for the prospective study is that the individuals are actively participating in organized sport and aged 10 years or older. Participants are excluded if they are medically unstable (e.g., active cardiac disease, progressive neurological disorder), have a peripheral or central nervous system disorder or ongoing musculoskeletal compromise of the upper extremity. For the current test-retest reliability studies, athletes between the ages of 18 and 40 years old who were participating in the larger prospective sample were recruited if they were healthy and without concussion within six months of study onset. Twenty-five athletes completed three assessments to evaluate test-retest reliability within a single season (*within-season* study). A separate group of 39 athletes completed assessments annually over three years (*year-to-year* study). All participants remained healthy and did not sustain any concussions over the course of the study periods. Visual acuity (Snellen chart, 20 feet) was determined in all 25 of the participants in the *within-season* study and 37 of the 39 individuals in the *year-to-year* study. Participants provided written, informed consent prior to assessments. Procedures were approved by the University of Calgary Conjoint Health Research Ethics Board (Ethics ID: 23963) and conducted in accordance with its guidelines.

### Robotic assessment

**Fig 1:**
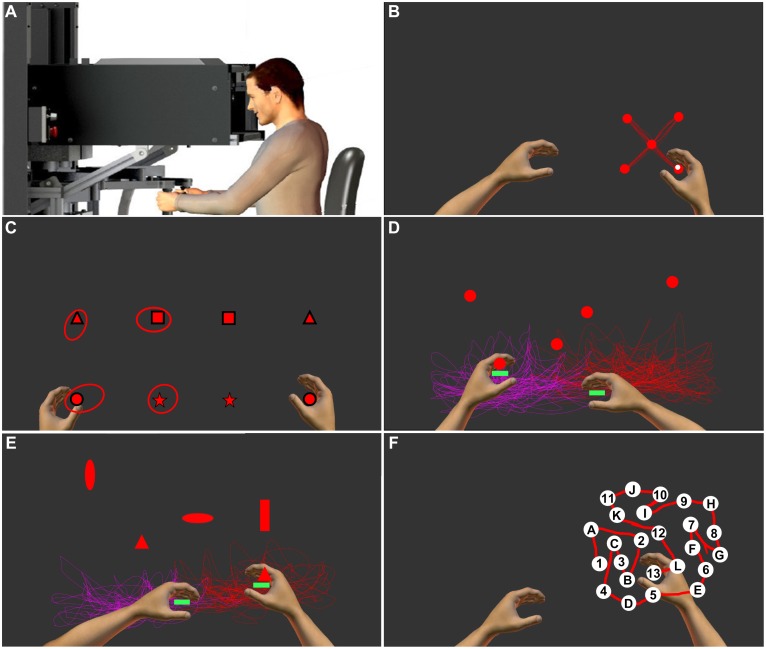
Depiction of the KINARM device and tasks. The KINARM end-point robotic device is shown in panel A and screen shots of a representative participant performing the tasks employed in the other panels (B, 4-target Visually guided reaching; C, 4-target Position Matching; D, Object Hit; E, Object Hit and Avoid; F, Trail Making B). In panel B, the red lines depict representative hand paths when moving to each target location. In panel C, the robot moved the dominant (passive) arm and the participant mirror-matched with the non-dominant (active) arm. Shapes represent each target location and the corresponding mirror matched position. Ellipses around the targets represent variability in position matching across trials. In panels D and E, red and purple lines depict dominant and non-dominant hand movements throughout the task. Green rectangles represent paddles that participants use to hit objects. In panel F, the red line depicts the participant’s hand path.

Robotic assessments were conducted with the KINARM end-point device. Seated in front of the KINARM and grasping the end-point handles, participants moved their arms in the horizontal plane to interact with a virtual reality system displayed in the same plane. Robot height was adjusted so participants’ heads rested in the centre of the visual field (Fig 1A). Participants completed three ~20 minute robotic assessments. *Within-season* study participants completed their first assessment (S1, i.e. session 1) during the pre-season, then a second assessment (S2) 1–3 months later, and their third assessment (S3) 9–12 months following S2. *Year-to-year* study participants completed Y1 (i.e. year 1), Y2 and Y3 assessments on an approximately annual basis in three consecutive athletic pre-seasons. No concussions were sustained between any of the assessments. Each assessment included the following tasks: Visually Guided Reaching [15,19,21], Position Matching [17,19,21,22], Object Hit [16], Object Hit and Avoid [14], and Trail making B [23,24].

The *Visually Guided Reaching (VGR)* (Fig 1B) task evaluated upper-extremity visuomotor capability. The robot handle was displayed as a white circle (0.5 cm radius) and targets shown as red circles (1.0 cm radius). Peripheral targets were presented discretely in a random order. Participants reached from the central target to one of four peripheral targets located 10 cm away and then back. Participants performed 20 out and 20 back reaching movements with the dominant arm as quickly and accurately as possible.

The *Position Matching (PM)* (Fig 1C) task assessed upper-extremity proprioception (position sense). The robot moved the dominant arm (passive arm) to one of four possible targets located at the corners of a 20 cm × 20 cm square grid. When the passive arm movement was complete, participants moved the non-dominant arm (active arm) to mirror-match the passive arm position, notifying the operator when they had matched to advance to the next trial. Vision of the arms and targets was occluded. Twenty-four trials were completed with target locations presented in a pseudo-random order.

The *Object Hit (OH)* (Fig 1D) task examined rapid, bimanual upper-extremity motor ability and visuospatial attention. Robot handles were represented as 2 cm wide green paddles. Objects (red circles with 2 cm radius) were “dropped” from 10 evenly spaced bins across the top of the screen. Participants used both hands to hit as many objects away as possible, receiving haptic feedback when an object was hit. Over 105 seconds, 30 objects were dropped at random from each bin (300 objects total). As the task progressed, object speed and frequency increased. The size of the paddles were smaller and the speed of the objects faster in this version of the task relative to the original task developed for the KINARM [16] because in pilot work the elite athletes demonstrated ceiling effects in performance on the original task.

The *Object Hit and Avoid (OHA)* (Fig 1E) task evaluated similar attributes as the OH task, with added emphasis on attention, rapid motor selection and inhibition. The task proceeded similarly to OH, except at the beginning participants were shown two of a possible ten object shapes that would be “dropped” from the bins at the top of the screen. Participants were instructed to hit those two “target” shapes, while avoiding the other eight “distractor” shapes. As with the OH task, participants received haptic feedback when contacting a target, but distractors passed through the participants’ paddle. Two hundred targets and 100 distractors were dropped over 105 seconds. Again, the size of the paddles were smaller and the speed of the objects faster in this version of the task relative to the original task developed for the KINARM [14].

The *Trail Making B (TMB)* (Fig 1F) task is a robot-based version of the standard paper and pencil task designed to evaluate visual attention and task-switching [23,24]. The robot handle was represented as a white circle (0.5 cm radius) and an array of 25 white circular targets labeled with letters (A-L) and numbers (1–13) was presented. Participants traced through the targets in an alpha-numeric sequence (i.e., 1-A-2-B-3-C…13-L). When a correct target was entered, the target turned green. If an incorrect target was entered, the target turned red and the participant was required to return to the prior correct target before continuing. Participants were familiarized to the task by completing a five-target version of the task prior to the full task. Participants were randomly presented with one of eight possible target patterns.

### Robotic outcome measures

Forty-four parameters quantified performance on spatial and temporal aspects of the five tasks (Table 1). Measures of performance on each parameter were quantified in each participant as a z-score relative to an age-corrected normative model of single session baseline performance in athletes between 18–40 years old who participated in the larger prospective study of SRC (VGR, n = 522, 351M/171F; PM, n = 520, 349M/171F; OH, n = 528, 355M/173F; OHA, n = 528, 356M/172F; TMB, n = 528, 356M/172F; BKIN Technologies Ltd.) [25]. Four athletes in the *within-season* test-retest reliability study were 17 years old at the time of their first assessment and were assigned z-scores as though they were 18 years old. All other participants were 18 years of age or older. Only parameters with a normally distributed dataset (or dataset that could be transformed to a normal distribution) are reported. Overall ‘Task Scores’ were also derived from a root-mean-square (RMS) distance of parameter z-scores for a given task (BKIN Technologies Ltd.), allowing consideration of overall performance on each task by combining performance on all task parameters into a single metric [25–27]. Task Scores near zero indicated superior performance and higher values indicated worse performance. A Task Score of one indicated performance was one standard deviation worse than the norm.

**Table 1:** Summary of KINARM tasks and parameters.

Task	Parameter	Definition	Behavioural attribute
Visually guided reaching, VGR (dominant hand)	Posture speed (m/s), PS	Mean hand speed when the hand should be at rest.	Upper limb postural control
	Reaction time (s), RT	Time from target onset to movement onset.	Motor response to a visual stimulus
	Initial direction error (°), IDE	Angular deviation between (i) a straight line from hand position at movement onset to destination target, (ii) a straight line from hand position at movement onset to hand position after initial phase of movement.	Feed-forward control: initial phase of movement.
	Initial distance ratio, IDR	Ratio of (i) distance hand travelled during initial phase of movement to (ii) distance hand travelled between movement onset and movement offset (or the end of the trial if the destination target is not reached).	Feed-forward control: initial phase of movement.
	Initial speed ratio, ISR	Ratio of (i) the maximum hand speed during initial phase of movement to (ii) global hand speed maximum of the trial.	Feed-forward control: initial phase of movement.
	Number of speed peaks, NSP	Number of hand speed maxima between movement onset and offset.	Feedback control: Movement corrections after initial motor response.
	Minimum maximum speed difference (m/s), MMSD	Differences between hand speed maxima and minima.	Feedback control: Movement corrections after initial motor response.
	Movement time (s), MT	Total time elapsed from movement onset to end.	Total movement.
	Path length ratio, PLR	Ratio of (i) distance travelled by hand between movement onset and movement offset and (ii) straight line distance between start and end targets.	Total movement.
	Maximum speed (m/s), MS	Global maximum hand speed.	Total movement.
Position matching, PM (dominant hand)	Variability XY (m), VarXY	Root-mean-square (RMS) of X and Y variables: mean value of variability of hand position in X and Y directions.	Position sense.
	Contraction expansion ratio, Cont/ExpXY	Ratio of range of area arm was moved by participant relative to arm moved by robot. Range of movement in X and Y directions are used in current ratio.	Position sense.
	Shift XY (m), ShiftXY	Mean difference between mirrored X and Y positions of the arm moved by participant and X and Y positions of arm moved by robot (+ lateral shift, -medial shift).	Position sense.
	Absolute error XY, AEXY	The mean absolute distance error in X and Y directions across all trials.	Position sense.
Object Hit, OH	Total hits, TH	Number of balls hit off screen in opposite direction from its original path.	Global performance.
	Hits dominant, HD	Number of balls hit with dominant hand.	Global performance.
	Hits non-dominant, HND	Number of balls hit with non-dominant hand.	Global performance.
	Hand bias hits, HBH	Value between -1 and 1 that describes the bias in number of balls hit by dominant and non-dominant hands.	Motor performance.
	Miss bias, MB	Value between -1 and 1 that describes the bias in number of balls missed in dominant and non-dominant sides of workspace.	Spatial performance.
	Hand transition, HT	Indicates where preference for using the dominant over non-dominant hand switches in work space.	Spatial performance.
	Hand selection overlap, HSO	Captures use of both hands and how often their use overlaps within workspace (i.e., balls hits with both dominant and non-dominant hands in same area of work space).	Motor performance.
	Median error, ME	Point in the task (% complete) when the participant made half of their errors.	Spatial and temporal performance.
	Hand speed dominant (m/s), HSD	Mean speed of dominant hand throughout the entire task.	Motor performance.
	Hand speed non-dominant (m/s), HSND	Mean speed of non-dominant hand throughout entire task.	Motor performance.
	Hand speed bias, HSB	Value between -1 and 1 that describes the bias in mean hand speed between the dominant and non-dominant hands.	Motor performance.
	Movement area dominant (m2), MAD	Area of space dominant hand entered during the entire task.	Motor performance.
	Movement area non-dominant (m2), MAND	Area of space the non-dominant hand entered during the entire task.	Motor performance.
	Movement area bias, MAB	A value from -1 to 1 that describes the bias in movement area between the dominant and non-dominant hands.	Motor performance.
Object Hit and Avoid, OHA	14 parameters from Object Hit +		
	Total distractor hits, TDH	Total number of distractor objects hit.	Global performance.
	Distractor hits dominant, DHD	Number of distractor objects hit with dominant hand.	Global performance.
	Distractor hits non-dominant, DHND	Number of distractor objects hit with non-dominant hand.	Global performance.
Trail Making B, TMB	Test time (s), TT	Total time from targets being shown to participant to touching the last target.	Executive function.
	Dwell time (s), DT	Total time spent with hand inside the targets.	Executive function.
	Time ratio, TR	Ratio of time to complete targets 13–25 to 1–12.	Executive function.
	Error count, EC	Number of times an incorrect target was touched.	Executive function.
Overall	Task score	Expresses participant’s performance as the number of standard deviations from the mean performance of a normative athlete group for each parameter. Derived from the RMS distance of a participant’s parameter z-scores for each task.	Overall performance of a task.

### Statistical analyses

Analyses were conducted separately for the *within-season* and *year-to-year* studies. ICCs evaluated test-retest reliability of the KINARM tasks between the first and second, and second and third assessments. The ICC model (2, 2) used a two-way repeated-measures, random effects analysis of variance model with type consistency. Assessment number was used as the random sample to compute the ICCs. ICCs of 0.75 and above, between 0.50 and 0.74, and below 0.50 were taken to indicate good, moderate, and poor reliability, respectively [12,28].

Bland-Altman (B-A) plots were inspected to identify systematic differences (i.e., practice effects) in measures between the first and second, and second and third assessments for each parameter. Creating a B-A plot involves plotting the mean of two measurements (e.g., assessment 1 and 2) against the difference between the same two measurements. When inspecting the B-A plots, the 95% limits of agreement, which describe the range of differences between the two measurements (mean±2SD), are commonly noted [28]. Specifically, B-A plots are used to describe the agreement between two quantitative measurements, rather than the relationship between them, as is achieved through correlational analyses [29]. When inspecting a B-A plot, the mean difference between the measurements is observed to determine potential bias or lack of agreement, and the relationship between the difference between the measurements and the true value (taken to be the mean of the two measurements) is considered. The bias can be considered significant if the confidence of the interval of the mean difference does not include the line of equality (i.e., zero) [29]. Thus, practice effects were considered present when the 95% confidence interval (CI) around the mean difference value did not cross zero.

Effect sizes described the magnitude of change, or practice effects, between assessments (S1 and S2, S2 and S3; Y1 and Y2, Y2 and Y3) for each measure [12,30]. Effect sizes were calculated as follows:

$$\delta =\frac{m2-m1}{SD1}$$

where:

δ = effect size.

*m1 =* group mean at baseline.

*m2 =* group mean at follow-up.

*SD1 =* group standard deviation at baseline.

For effect size calculations between the second and third assessment, the second and third assessments were considered baseline and follow-up, respectively. Effect sizes with absolute values of 0.80 and above, between 0.50 and 0.79, and below 0.50 were considered large, medium and small, respectively [31]. Statistical analyses were performed in MATLAB 2016a (Mathworks, Natick, MA, USA). ICCs were calculated using the Intraclass Correlation Coefficient function [32].

## Results

### Participants

Athletes in the *within-season* study were all male, 20.2±2.1 (mean±SD) years of age, and had visual acuity of 20/50 uncorrected or greater. The *year-to-year* study participants were an average of 22.8±6.0 years of age (28M, 11F) and had visual acuity of 20/40 uncorrected or greater. Participants who typically wore corrective lenses also wore them during the robotic testing on both initial and repeat assessments. Participant characteristics are highlighted in Table 2.

**Table 2:** Participant characteristics.

	Within-Season	Year-to-year
**n**	25	39
**Age at Assessment 1 (years)**	20.2±2.2	22.8±6.0
**Sex**	25M, 0F	28M, 11F
**Dominant hand**	24R, 1L	35R, 4L
**History of concussion**	0 concussions: n = 16	0 concussions: n = 17
	1 concussion: n = 8	1 concussion: n = 12
	*unknown: n = 1	2 concussions: n = 7
		≥ 3 concussions: n = 3
**Time from Assessment 1 to Assessment 2 (days)**	47.8±32.7	361.0±50.1
**Time from Assessment 2 to Assessment 3 (days)**	316.1±32.7	319.7±66.1
**Sport**	18 Varsity Football	16 Varsity Football
	7 Varsity Men’s Hockey	11 National Skeleton
		6 National Luge
		3 National Bobsled
		3 National Alpine

M, male; F, female; R, right; L, left; While many individuals had a history of concussion, all were asymptomatic at the time of all and none sustained a concussion over the course of the studies.

*One individual was missing information on history of concussion.

### Within-season test-retest reliability

**Fig 2:**
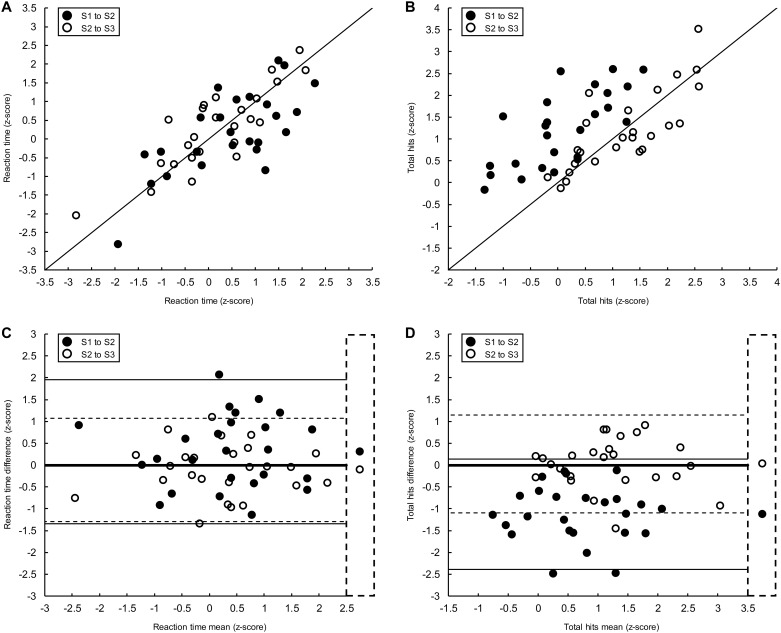
Scatter plots (top) and B-A plots (bottom) for representative parameters in the VGR (left) and OH (right) tasks from the *within-season* test-retest reliability study. On scatter plots (A and B), filled markers represent measures taken at S1 (x-axis) and S2 (y-axis). Open markers represent measures taken at S2 (x-axis) and S3 (y-axis). The black line depicts the unity line. On B-A plots, the values depicted on the x- and y-axis were derived from measures taken at S1 and S2 (filled markers), and S2 and S3 (open markers). Horizontal lines show 95% limits of agreement (S1 to S2, solid line; S2 and S3, dashed line). The thick black horizontal lines denote no difference (zero, line of equality) in performance between assessments. Markers in the dashed line box represent mean differences in performance between assessments.

Fig 2 shows data from representative parameters in the VGR (A, C) and OH tasks (B, D). There was strong correlation between measurements taken across assessments in VGR Reaction Time (ICC_S1-S2_ = 0.83 [95% CI: 0.61–0.93]; ICC_S2toS3_ = 0.91 [95% CI: 0.81–0.96]). Even distribution of data points around the unity line suggested no practice effects for this parameter. Inspection of the B-A plot (Fig 2C) also suggested no practice effects between assessments (S1 to S2, S2 to S3), as Reaction Time difference values were evenly and randomly distributed around zero (δ_S1toS2_ = 0.28; δ_S2toS3_ = -0.10). OH Total Hits (Fig 2B) also had strong correlation between measurements taken across assessments (ICC_S1-S2_ = 0.83 [95% CI: 0.61–0.93]; ICC_S2toS3_ = 0.88 [95% CI: 0.76–0.95]); however, data points showing S1 and S2 measurements (filled markers) were all distributed above the unity line, suggesting a S1-to-S2 practice effect. A practice effect was not observed between S2 and S3 (open markers). These observations were corroborated by the B-A plot (Fig 2D), which shows that data points depicting the difference in Total Hits between S1 and S2 (filled markers) were all below the line of equality, while differences in Total Hits between S2 and S3 (open markers) were evenly and randomly distributed around zero (δ_S1toS2_ = -1.35; δ_S2toS3_ = 0.03). While the majority of parameters did not show large practice effects, those that did (e.g., Total Hits from the OH task) only changed markedly between S1 and S2, with performance leveling between S2 and S3 (Table 3).

**Table 3:** *Within-season* study results.

Task and parameters	Reliability		Agreement (B-A)		Effect size	
	ICC (CI)		Mean difference (95% limits)		δ	
	S1-S2	S2-S3	S1-S2	S2-S3	S1-S2	S2-S3
**Visually Guided Reaching Parameters**						
Posture speed (m/s)	0.61 (0.11, 0.83)*	0.39 (-0.38, 0.73)	0.31 (-1.80, 2.41)	0.06 (-2.11, 2.23)	0.31	0.05
Reaction time (s)	0.83 (0.61, 0.93)**	0.91 (0.81, 0.96)**	0.31 (-1.34, 1.95)	-0.11 (-1.29, 1.08)	0.28	-0.10
Initial direction error (rad)	0.72 (0.36, 0.88)*	0.80 (0.54, 0.91)**	0.91 (-1.50, 3.31)	0.20 (-1.77, 2.18)	0.63	0.17
Initial distance ratio	0.84 (0.64, 0.93)**	0.86 (0.68, 0.94)**	-0.66 (-2.38, 1.06)	-0.17 (-1.79, 1.46)	-0.50	-0.16
Speed maxima count	0.80 (0.54, 0.91)**	0.54 (-0.04, 0.80)*	0.28 (-1.57, 2.13)	0.13 (-2.47, 2.73)	0.22	0.13
Min-max speed difference (m/s)	0.81 (0.56, 0.91)**	0.89 (0.74, 0.95)**	0.45 (-1.39, 2.29)	0.20 (-1.39, 1.79)	0.39	0.17
Movement time (s)	0.42 (-0.31, 0.74)	0.60 (0.10, 0.83)*	-0.08 (-2.48, 2.32)	-0.10 (-2.35, 2.15)	-0.08	-0.09
Path length ratio	0.73 (0.39, 0.88)*	0.90 (0.77, 0.96)**	0.49 (-1.33,2.31)	0.05 (-1.23, 1.33)	0.47	0.05
Maximum speed (m/s)	0.72 (0.37, 0.88)*	0.88 (0.74, 0.95)**	0.33 (-1.53, 2.20)	(-1.52, 1.29)	0.34	-0.11
Task score	0.53 (-0.07, 0.79)*	0.92 (0.81, 0.96)**	0.38 (-1.17, 1.94)	-0.03 (-0.069, 0.63)	0.48	-0.05
**Arm Position Matching Parameters**						
Variability XY (m)	0.41 (-0.33, 0.74)	-0.07 (-1.43, 0.53)	0.36 (-1.66, 2.38)	-0.09 (-2.59, 2.41)	0.47	-0.10
Contraction/expansion ratio XY	0.62 (0.15, 0.83)*	0.76 (0.46, 0.90)**	-0.05 (-1.86, 1.76)	0.26 (-1.41, 1.93)	-0.07	0.26
Shift XY (m)	0.42 (-0.31, 0.75)	0.66 (0.24, 0.85)*	-0.24 (-2.40, 1.92)	0.24 (-1.73, 2.21)	-0.31	0.24
Absolute error XY	0.30 (-0.58, 0.69)	0.57 (0.03, 0.81)*	-0.37 (-2.58, 1.84)	0.32 (-1.60, 2.24)	-0.42	0.36
Task score	0.52 (-0.09, 0.79)*	0.58 (0.05, 0.81)*	-0.09 (-1.19, 1.02)	0.09 (-0.97, 1.15)	-0.16	0.21
**Object Hit Parameters**						
Total hits	0.83 (0.61, 0.93)**	0.88 (0.76, 0.95)**	-1.12 (-2.39, 0.14)	0.03 (-1.09, 1.15)	-1.35ϯ	0.03
Hits dom	0.59 (0.06, 0.82)*	0.83 (0.63, 0.93)**	-0.86 (-2.51, 0.79)	0.10 (-1.15, 1.35)	-1.36ϯ	0.12
Hits non-dom	0.85 (0.66, 0.93)**	0.85 (0.67, 0.94)**	-0.70 (-1.98, 0.59)	-0.04 (-1.22, 1.14)	-0.74	-0.05
Hand bias hits	0.65 (0.20, 0.85)*	0.81 (0.57, 0.92)**	-0.07 (-1.71, 1.57)	0.12 (-1.21, 1.45)	-0.10	0.13
Miss bias	0.41 (-0.34, 0.74)	0.00 (-1.28, 0.56)	0.01 (-2.69, 2.71)	0.14 (-3.23, 3.51)	0.01	0.12
Hand transition	0.54 (-0.04, 0.80)*	0.61 (0.11, 0.83)*	0.13 (-2.23, 2.48)	-0.34 (-2.26, 1.58)	0.12	-0.32
Hand selection overlap	0.53 (-0.08, 0.79)*	0.41 (-0.34, 0.74)	-0.18 (-2.39, 2.04)	0.27 (-2.12, 2.65)	-0.20	0.25
Median error	0.31 (-0.57, 0.69)	0.51 (-0.10, 0.79)*	-1.13 (-3.19, 0.94)	0.28 (-1.59, 2.14)	-1.36ϯ	0.33
Hand speed dom (m/s)	0.90 (0.76, 0.95)**	0.64 (0.18, 0.84)*	-0.41 (-1.66, 0.84)	0.09 (-1.75, 1.93)	-0.40	0.09
Hand speed non-dom (m/s)	0.93 (0.85, 0.97)**	0.59 (0.07, 0.82)*	-0.23 (-1.20, 0.74)	0.01 (-1.76, 1.78)	-0.23	0.01
Hand speed bias	0.64 (0.18, 0.84)*	0.71 (0.35, 0.87)*	-0.27 (-2.18, 1.63)	0.13 (-1.42, 1.68)	-0.26	0.16
Movement area dom (m^2^)	0.89 (0.75, 0.95)**	0.71 (0.35, 0.87)*	-0.63 (-1.91, 0.66)	0.22 (-1.62, 2.06)	-0.65	0.20
Movement area non-dom (m^2^)	0.83 (0.61, 0.93)**	0.73 (0.38, 0.88)*	-0.54 (-2.23, 1.15)	0.08 (-1.77, 1.94)	-0.47	0.07
Movement area bias	0.59 (0.06, 0.82)*	0.54 (-0.05, 0.80)*	-0.14 (-2.57, 2.30)	0.27 (-2.19, 2.73)	-0.14	0.20
Task score	0.44 (-0.28, 0.75)	0.38 (-0.40, 0.73)	0.19 (1.06, 1.43)	0.12 (-1.15, 1.40)	0.41	0.20
**Object Hit and Avoid Parameters**						
Total hits	0.72 (0.37, 0.88)*	0.76 (0.46, 0.90)**	-0.23 (-2.14, 1.69)	-0.16 (-2.15, 1.83)	-0.27	-0.13
Hits dom	0.28 (-0.6, 0.68)	0.73 (0.38, 0.88)*	-0.09 (-2.67, 2.49)	-0.38 (-2.27, 1.50)	-0.09	-0.35
Hits non-dom	0.59 (0.07, 0.82)*	0.65 (0.21, 0.85)*	-0.39 (-2.61, 1.83)	0.24 (-1.98, 2.45)	-0.44	0.20
Hand bias hits	-0.09 (-1.47, 0.52)	0.42 (-0.31, 0.75)	0.22 (-2.53, 2.97)	-0.46 (-2.45, 1.54)	0.23	-0.47
Miss bias	0.07 (-1.11, 0.59)	0.56 (0.00, 0.81)*	-0.13 (-2.67, 2.40)	0.35 (-1.77, 2.46)	-0.12	0.45
Hand transition	0.32 (-0.55, 0.70)	0.56 (0.00, 0.81)*	-0.07 (-2.51, 2.38)	0.24 (-1.67, 2.15)	-0.07	0.24
Hand selection overlap	0.59 (0.08, 0.82)*	0.59 (0.07, 0.82)*	0.13 (-1.99, 2.24)	0.20 (-2.02, 2.43)	0.12	0.21
Median error	0.40 (-0.37, 0.73)	0.45 (-0.24, 0.76)	0.24 (-2.14, 2.63)	-0.21 (-2.71, 2.30)	0.28	-0.19
Hand speed dom (m/s)	0.76 (0.45, 0.89)**	0.81 (0.57, 0.92)**	0.42 (-1.46, 2.30)	-0.27 (-1.74, 1.20)	0.37	-0.26
Hand speed non-dom (m/s)	0.81 (0.56, 0.91)**	0.67 (0.24, 0.85)*	0.30 (-1.28, 1.88)	-0.06 (-1.78, 1.67)	0.27	-0.06
Hand speed bias	0.16 (-0.90, 0.63)	0.41 (-0.35, 0.74)	0.17 (-2.56, 2.89)	-0.33 (-2.43, 1.78)	0.16	-0.32
Movement area dom (m^2^)	0.79 (0.52, 0.91)**	0.84 (0.64, 0.93)**	-0.02 (-1.83, 1.78)	-0.15 (-1.51, 1.21)	-0.02	-0.17
Movement area non-dom (m^2^)	0.84 (0.64, 0.93)**	0.69 (0.30, 0.86)*	-0.18 (-1.77, 1.40)	0.26 (-1.71, 2.24)	-0.16	0.25
Movement area bias	0.68 (0.28, 0.86)*	0.23 (-0.75, 0.66)	0.12 (-1.93, 2.18)	-0.47 (-3.26, 2.32)	0.14	-0.39
Task score	0.75 (0.44, 0.89)**	0.48 (-0.18, 0.77)	-0.19 (-1.26, 0.88)	0.12 (-1.22, 1.46)	-0.36	0.17
**Trail Making B Parameters**						
Test time	0.30 (-0.59, 0.69)	0.56 (0.00, 0.81)*	1.04 (-1.28, 3.36)	-0.42 (-2.51, 1.66)	1.22ϯ	-0.43
Dwell time	0.72 (0.37, 0.88)*	0.68 (0.28, 0.86)*	0.79 (-0.99, 2.57)	-0.08 (-1.76, 1.59)	0.80ϯ	-0.09
Time ratio	0.17 (-0.89, 0.63)	-0.20 (-1.72, 0.47)	-0.35 (-3.03, 2.33)	0.27 (-2.78, 3.33)	-0.30	0.34
Task score	0.32 (-0.54,0.70)	0.58 (0.04, 0.81)*	0.51 (-0.58, 1.59)	-0.17 (-1.11, 0.76)	1.02ϯ	-0.47

S1, session 1; S2, session 2; S3, session 3. ICC values suggesting moderate (0.50 to 0.74)* and good reliability (≥ 0.75)** are denoted by asterisk symbols. Effect size (δ) values with a large practice effect (≥0.80) are denoted by cross symbols (ϯ). Five additional parameters not listed in this table were collected and have been described in prior work (VGR: initial speed ratio [7]; OHA: Distractor hits total, dominant and non-dominant [6]; TMB: Error count [11,12]), but data from these parameters could not be transformed to fit a normal distribution and thus, a normative model could not be computed to determine z-scores. As a result, these parameters are not reported here.

Results from all analyses conducted for the *Within-season* study are presented in Table 3. Considering performance in S1 and S2, ICCs were below 0.50 for 32% of parameters, between 0.50 and 0.74 for 38% of parameters, and 0.75 or greater for 30% of parameters. Between S2 and S3, ICCs were below 0.50 for 20% of parameters, between 0.50 and 0.74 for 48% of parameters, and 0.75 or greater for 32% of parameters. B-A plots suggested agreement between assessments in most parameters, with practice effects apparent in 45% of parameters between S1 and S2 and 18% of parameters between S2 and S3. Examination of δ values suggested only small practice effects for the majority of parameters. Between S1 and S2, δ values were below 0.50 for 80% of parameters, between 0.50 and 0.79 for 9% of parameters and 0.80 or greater for 11% of parameters. The only parameters for which large δ values ≥0.80 were found between S1 and S2 were Total Hits, Hits with Dominant Hand and Median Error in the OH task, and Test Time and Dwell Time in the TMB task. Between S2 and S3, practice effects dissipated and δ values were small (below 0.50) for all parameters.

Overall Task Scores yielded ICCs greater than 0.50 for VGR, PM, and OHA between S1 and S2, and for VGR, PM and TMB between S2 and S3. Only the TMB Task Score had a δ of 0.80 or higher and only between S1 and S2.

### Year-to-year test-retest reliability

**Fig 3:**
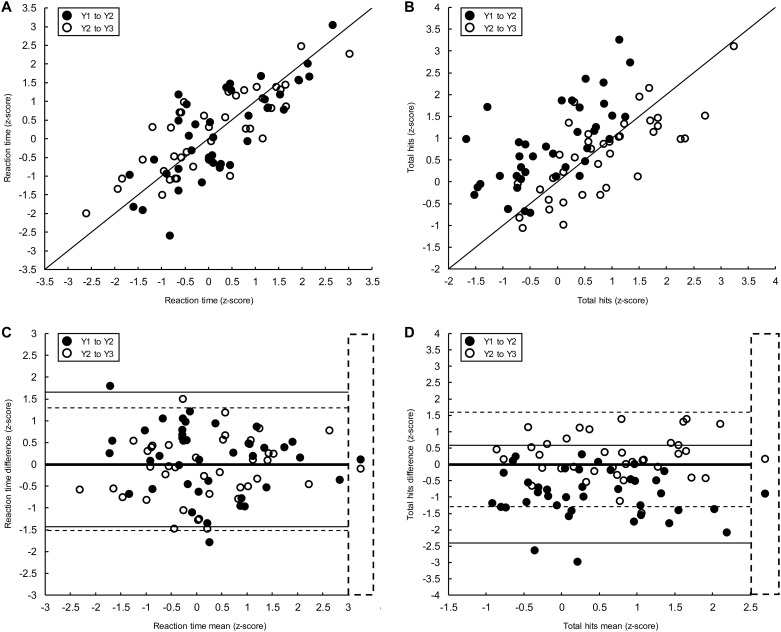
Scatter plots (top) and B-A plots (bottom) for representative parameters in the VGR (left) and OH (right) tasks from the *year-to-year* test-retest reliability study. On scatter plots (A and B), filled markers represent measures taken at Y1 (x-axis) and Y2 (y-axis). Open markers represent measures taken at Y2 (x-axis) and Y3 (y-axis). The black line depicts the unity line. On B-A plots, the values depicted on the x- and y- axis were derived from measures taken at Y1 and Y2 (filled markers), and Y2 and Y3 (open markers). Horizontal lines show 95% limits of agreement (Y1 to Y2, solid line; Y2 and Y3, dashed line). The thick black horizontal lines denote no difference (zero, line of equality) in performance between assessments. Markers in the dashed line box represent mean differences in performance between assessments.

Fig 3 shows data from representative parameters in the VGR (A, C) and OH tasks (B, D). Similar to the *within-season* study, there was strong correlation between VGR Reaction Time measurements taken between assessments (Fig 3A, ICC_Y1-Y2_ = 0.86 [95% CI: 0.74–0.93]; ICC_Y2toY3_ = 0.89 [95% CI: 0.79–0.94]), good agreement (Fig 3C) and small practice effect sizes (δ_Y1toY2_ = 0.11; δ_Y2toY3_ = -0.09). OH Total Hits also showed a strong correlation between measurements taken across assessments (Fig 3B, ICC_Y1-Y2_ = 0.83 [95% CI: 0.61–0.93]; ICC_Y2toY3_ = 0.88 [95% CI: 0.76, 0.95]); however, most data points showing Y1 and Y2 measurements (filled markers) fell above the line of unity and difference values between Y1 and Y2 depicted on the B-A plot (Fig 3D) were mostly below the line of equality. This evidence of a practice effect dissipated between Y2 and Y3 (open markers, Fig 3B and 3D), as was also reflected in the effect size values (δ_Y1toY2_ = -1.10; δ_Y2toY3_ = 0.21). Similar to the *within-season* data, Total Hits from the OH task is one of only a few parameters (three total) that showed a substantial practice effect ≥0.80 between Y1 and Y2, and performance then plateaued between Y2 and Y3.

*Year-to-year* study results are presented in Table 4. Considering performance in Y1 and Y2, ICCs were below 0.50 for 36% of parameters, between 0.50 and 0.74 for 43% of parameters, and 0.75 or above for 21% of parameters. Between Y2 and Y3, ICCs were below 0.50 for 18% of parameters, between 0.50 and 0.74 for 34% of parameters, and 0.75 or above for 48% of parameters. B-A plots suggested agreement between assessments in most parameters, with practice effects apparent in 34% of parameters between Y1 and Y2, and 16% of parameters between Y2 and Y3. Examination of practice effect sizes supported these observations. Between Y1 and Y2, practice effect sizes were below 0.50 for 86% of parameters, between 0.50 and 0.79 for 7% of parameters and 0.80 or above for 7% of parameters. The only parameters for which large δ values ≥0.80 were Total Hits, Hits with Non-dominant Hand and Median Error in the OH task between Y1 and Y2. Between Y2 and Y3, performance plateaued with δ values below 0.50 for all parameters.

**Table 4:** *Year-to-year* study results.

Task and parameters	Reliability		Agreement (B-A)		Effect size	
	ICC (CI)		Mean difference (95% limits)		δ	
	Y1-Y2	Y2-Y3	Y1-Y2	Y2-Y3	Y1-Y2	Y2-Y3
**Visually Guided Reaching**						
Posture speed (m/s)	0.62 (0.27, 0.80)*	0.81 (0.63, 0.90)**	-0.14 (-2.23, 1.94)	0.22 (-1.43, 1.88)	-0.15	0.21
Reaction time (s)	0.86 (0.74, 0.93)**	0.89 (0.79, 0.94)**	0.11 (-1.43, 1.66)	-0.11 (-1.52, 1.30)	0.11	-0.09
Initial direction error (rad)	0.81 (0.63, 0.90)**	0.74 (0.51, 0.86)*	0.26 (-1.67, 2.20)	0.08 (-2.00, 2.16)	0.21	0.07
Initial distance ratio	0.66 (0.35, 0.82)*	0.71 (0.45, 0.85)*	-0.47 (-2.77, 1.83)	-0.13 (-2.26, 2.01)	-0.41	-0.11
Speed maxima count	0.38 (-0.19, 0.67)	0.62 (0.28, 0.80)*	0.29 (-2.49, 3.06)	0.14 (-2.24, 2.52)	0.24	0.13
Min-max speed difference (m/s)	0.87 (0.75, 0.93)**	0.89 (0.79, 0.94)**	0.32 (-1.34, 1.97)	0.23 (-1.46, 1.91)	0.27	0.17
Movement time (s)	0.76 (0.54, 0.87)**	0.79 (0.61, 0.89)**	0.19 (-1.70, 2.09)	-0.08 (-1.70, 1.53)	0.17	-0.08
Path length ratio	0.85 (0.72, 0.92)**	0.88 (0.77, 0.94)**	0.26 (-1.44, 1.96)	0.17 (-1.43, 1.76)	0.24	0.13
Maximum speed (m/s)	0.92 (0.85, 0.96)**	0.92 (0.84, 0.96)**	0.01 (-1.19, 1.21)	0.06 (-1.23, 1.35)	0.01	0.05
Task score	0.74 (0.51, 0.86)*	0.74 (0.50, 0.86)*	0.08 (-1.19, 1.36)	0.12 (-1.00, 1.24)	0.12	0.17
**Arm Position Matching**						
Variability XY (m)	0.47 (-0.02, 0.72)	0.67 (0.36, 0.83)*	0.41 (-.23, 3.04)	0.39 (-1.59, 2.36)	0.33	0.37
Contraction/expansion ratio XY	0.60 (0.23, 0.79)*	0.78 (0.58, 0.88)**	0.19 (-1.79, 2.17)	0.12 (-1.23, 1.47)	0.18	0.15
Shift XY (m)	0.27 (-0.39, 0.62)	0.54 (0.12, 0.76)*	0.06 (-2.40, 2.52)	-0.16 (-2.10, 1.78)	0.06	-0.18
Absolute error XY	0.48 (0.01, 0.73)	0.61 (0.25, 0.79)*	0.26 (-1.98, 2.51)	0.00 (-2.05, 2.05)	0.27	0.00
Task score	0.59 (0.22, 0.79)*	0.65 (0.32, 0.81)*	0.35 (-1.03, 1.72)	-0.05 (-1.01, 0.91)	0.44	-0.11
**Object Hit**						
Total hits	0.78 (0.59, 0.89)**	0.86 (0.72, 0.92)**	-0.93 (-2.41, 0.54)	0.20 (-1.10, 1.51)	-1.10ϯ	0.21
Hits dom	0.33 (-0.28, 0.65)	0.74 (0.51, 0.86)*	-0.45 (-2.68, 1.78)	0.17 (-1.42, 1.77)	-0.48	0.20
Hits non-dom	0.67 (0.37, 0.83)*	0.86 (0.73, 0.92)**	-0.88 (-2.52, 0.77)	0.11 (-1.25, 1.47)	-1.12ϯ	0.12
Hand bias hits	-0.09 (-1.08, 0.43)	0.76 (0.54, 0.87)**	0.40 (-2.20, 3.00)	-0.04 (-2.08, 2.01)	0.45	-0.03
Miss bias	0.67 (0.36, 0.82)*	0.71 (0.45, 0.85)*	-0.10 (-2.56, 2.36)	-0.02 (-2.48, 2.45)	-0.09	-0.01
Hand transition	0.52 (0.09, 0.75)*	0.65 (0.34, 0.82)*	0.04 (-2.55, 2.63)	-0.18 (-2.35, 2.00)	0.03	-0.17
Hand selection overlap	0.46 (-0.04, 0.72)	0.67 (0.38, 0.83)*	-0.17 (-2.67, 2.34)	0.57 (-1.34, 2.49)	-0.17	0.49
Median error	0.14 (-0.64, 0.55)	0.34 (-0.26, 0.65)	-0.84 (-3.20, 1.52)	0.02 (-1.85, 1.89)	-0.82ϯ	0.03
Hand speed dom (m/s)	0.63 (0.29, 0.80)*	0.87 (0.75, 0.93)**	-0.25 (-2.02, 1.52)	0.14 (-0.92, 1.20)	-0.27	0.17
Hand speed non-dom (m/s)	0.65 (0.34, 0.82)*	0.89 (0.79, 0.94)**	-0.39 (-2.03, 1.25)	0.10 (-0.99, 1.20)	-0.51	0.12
Hand speed bias	0.43 (-0.10, 0.70)	0.76 (0.54, 0.87)**	0.20 (-1.78, 2.19)	0.04 (-1.57, 1.66)	0.26	0.05
Movement area dom (m^2^)	0.49 (0.02, 0.73)	0.83 (0.68, 0.91)**	-0.21 (-2.21, 1.80)	-0.05 (-1.18, 1.09)	-0.20	-0.06
Movement area non-dom (m^2^)	0.53 (0.10, 0.75)*	0.75 (0.53, 0.87)**	-0.29 (-2.24, 1.66)	0.05 (-1.42, 1.51)	-0.31	0.06
Movement area bias	0.66 (0.36, 0.82)*	0.32 (-0.30, 0.64)	0.14 (-1.81, 2.09)	-0.14 (-2.63, 2.35)	0.14	-0.13
Task score	0.68 (0.39, 0.83)*	0.66 (0.35, 0.82)*	0.13 (-0.88, 1.15)	0.07 (-1.03, 1.11)	0.24	0.08
**Object Hit and Avoid**						
Total hits	0.57 (0.18, 0.77)*	0.76 (0.55, 0.88)**	-0.39 (-2.51, 1.72)	-0.16 (-1.79, 1.47)	-0.36	-0.18
Hits dom	0.37 (-0.19, 0.67)	0.66 (0.36, 0.82)*	-0.23 (-2.59, 2.13)	0.03 (1.80, 1.86)	-0.23	0.03
Hits non-dom	0.69 (0.40, 0.84)*	0.79 (0.59, 0.89)**	-0.36 (-2.25, 1.53)	-0.26 (-1.75, 1.24)	-0.33	-0.29
Hand bias hits	0.55 (0.15, 0.77)*	0.67 (0.38, 0.83)*	0.14 (1.97, 2.25)	0.22 (-1.28, 1.82)	0.13	0.25
Miss bias	0.20 (-0.52, 0.58)	0.07 (-0.77, 0.51)	0.01 (-2.66, 2.68)	0.03 (-2.85, 2.91)	0.02	0.03
Hand transition	0.48 (0.01, 0.73)	0.17 (-0.59, 0.56)	-0.01 (-2.36, 2.33)	-0.28 (-2.69, 2.13)	-0.01	-0.31
Hand selection overlap	-0.07 (-1.04, 0.44)	0.11 (-0.69, 0.53)	0.22 (-2.34, 2.78)	-0.14 (-2.66, 2.37)	0.22	-0.17
Median error	-0.14 (-1.17, 0.40)	0.01 (-0.90, 0.48)	-0.20 (-3.14, 2.73)	0.06 (-2.67, 2.80)	-0.20	0.06
Hand speed dom (m/s)	0.68 (0.39, 0.83)*	0.80 (0.62, 0.90)**	-0.12 (-1.78, 1.53)	0.13 (-1.31, 1.58)	-0.15	0.15
Hand speed non-dom (m/s)	0.72 (0.47, 0.86)*	0.91 (0.83, 0.95)**	-0.16 (-1.75, 1.43)	0.01 (-1.09, 1.11)	-0.21	0.01
Hand speed bias	0.61 (0.26, 0.80)*	0.67 (0.37, 0.83)*	0.00 (-2.02, 2.01)	0.14 (-1.48, 1.77)	0.00	0.15
Movement area dom (m^2^)	0.75 (0.52, 0.87)**	0.72 (0.47, 0.85)*	-0.14 (-1.59, 1.31)	-0.06 (-1.66, 1.55)	-0.17	-0.07
Movement area non-dom (m^2^)	0.58 (0.21, 0.78)*	0.69 (0.40, 0.84)*	-0.06 (-1.83, 1.70)	-0.16 (-1.99, 1.67)	-0.09	-0.17
Movement area bias	0.63 (0.29, 0.80)*	0.38 (-0.18, 0.68)	-0.16 (-2.38, 2.06)	0.15 (-2.38, 2.69)	-0.16	0.14
Task score	0.32 (-0.30, 0.64)	0.41 (-0.13, 0.69)	0.08 (-1.63, 1.79)	0.07 (-1.33, 1.48)	0.12	0.11
**Trail Making B**						
Test time	0.70 (0.42, 0.84)*	0.77 (0.56, 0.88)**	0.52 (-1.27, 2.32)	0.18 (-1.57, 1.94)	0.65	0.17
Dwell time	0.78 (0.59, 0.89)**	0.86 (0.74, 0.93)**	0.63 (-1.05, 2.31)	0.20 (-1.34, 1.73)	0.69	0.18
Time ratio	0.16 (-0.60, 0.56)	0.40 (-0.14, 0.69)	-0.08 (-2.54, 2.38)	0.21 (-1.94, 2.36)	-0.09	0.22
Task score	0.75 (0.53, 0.87)**	0.73 (0.49, 0.86)*	0.25 (-0.79, 1.28)	0.12 (-0.93, 1.16)	0.44	0.19

Y1, year 1; Y2, year 2; Y3, year 3. ICC values suggesting moderate (0.50 to 0.74)* and good reliability (≥ 0.75)** are denoted by asterisk symbols. Effect size (δ) values with a large practice effect (≥0.80) are denoted by cross symbols (ϯ). Five additional parameters not listed in this table were collected and have been described in prior work (VGR: initial speed ratio [7]; OHA: Distractor hits total, dominant and non-dominant [6]; TMB: Error count [11,12]), but data from these parameters could not be transformed to fit a normal distribution and thus, a normative model could not be computed to determine z-scores. As a result, these parameters are not reported here.

Overall Task Scores yielded ICCs greater than 0.50 for all but the OHA task between both sets of time points (Y1 to Y2, and Y2 to Y3), and practice effects were minimal for all tasks between each time point (all δ’s <0.50).

**Fig 4:**
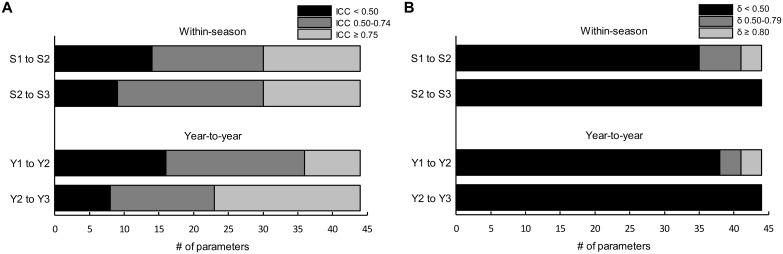
Summary of ICCs (A) and δ values (B) across *within-season* and *year-to-year* test-retest reliability studies. Panel A depicts the number of parameters (/44) with ICCs <0.50 (poor, black bar), between 0.50–0.74 (moderate, dark grey bar) and ≥0.75 (good, light grey bar). Panel B depicts the number of parameters (/44) with δ values <0.50 (small, black bar), between 0.50–0.79 (moderate, dark grey bar) and ≥0.80 (large, light grey bar).

A summary of all ICC and effect size results for both the *within-season* and *year-to-year* studies is presented in Fig 4.

## Discussion

The main finding of the current work was that there was moderate-to-good test-retest reliability for most performance parameters derived from the KINARM. Large practice effects were apparent between the first and second assessment for a minority of parameters (20% in *within-season*, 14% in *year-to-year*), with no considerable performance changes made between a second and third assessment for any parameters. These findings were comparable whether assessments were conducted within a single season or in the pre-season period for three consecutive seasons.

A study of pediatric ice-hockey players employed similar analyses of the same KINARM assessments as those included in the present work, but conducted two assessments in immediate succession on the same day, and a third assessment one week later [12]. In the pediatric study [12] and the current *within-season* and *year-to year* studies conducted in adults, ICCs were 0.50 or above for the majority of the parameters across all assessments. Importantly, our current results suggest that if an individual were assessed at a baseline time point and then re-assessed after sustaining a SRC, the reliability of the majority of the KINARM parameters would be expected to be similar whether the injury occurred early (i.e. *within-season study* results) or late in the season (i.e. *year-to-year* results). Nevertheless, the proportion of parameters with moderate-to-good reliability between the first and second assessment appeared higher in the pediatric study (75%) [12] compared to the present work (*within-season study*, 68%’ *year-to-year study*, 64%). Also, in the pediatric study there was little difference in the proportion of parameters with ICCs above 0.50 between assessments (75% Assessment 1 to 2, 73% Assessment 2 to 3) [12]. In contrast, for both the *within-season* and *year-to-year* studies there were more parameters with ICCs above 0.50 between the second and third, relative to the first and second assessments (*within-season*, 68% S1 to S2, 80% S2 to S3; *year-to-year*, 64% Y1 to Y2, 82% Y2 to Y3). These slightly disparate findings likely relate to the short interval between tests in the pediatric study [12] relative to the studies reported here. Regardless, moderate-to-good reliability was found for the majority of KINARM task performance parameters previously in pediatric athletes [12], and now here in young adult athletes across two timeframes of testing.

The test-retest reliability of the subset of KINARM Standard Tests studied presently appears comparable to other assessment tools currently being used to evaluate SRC in clinical and research settings. For example, investigation of test-retest reliability of computerized neurocognitive tests, such as the ImPACT, in athletes has yielded results suggesting a range from very poor to very good reliability [9,33–35]. A study of young healthy individuals completing the Sensory Organization Test of Computerized Dynamic Posturography (SOT), a test commonly used to evaluate postural stability in SRC research [36,37], determined an ICC of 0.64 for its composite score and ICCs ranging from 0.43 to 0.79 for the scores derived for each of the six SOT conditions [38]. Also, a test-retest reliability study of the post-concussion symptom scale in the Sport Concussion Assessment Tool (version 3) reported an ICC of 0.43 at an approximately 6-month test interval [39]. Additionally, the Test Time parameter from the robot-based version of the Trails B test used currently yielded an ICC of 0.30 in the *within-season* study and 0.70 in the *year-to-year* study when considering the first and second assessments. Prior work with the pencil and paper version of this task has reported ICCs ranging from 0.39 to 0.85 [33,40,41]. The higher ICC in the *year-to-year* study relative to the *within-season* study here likely relates to an almost two-fold smaller practice effect in the *year-to-year* study for this particular parameter (*within-season* δ = 1.22; *year-to-year* δ = 0.65).

When evaluating practice effects in this study, only a small proportion of parameters demonstrated a large change in performance between the first and second assessments (*within-season*, 11%; *year-to-year*, 7%). In the *within-season* study, these large practice effects were exclusively observed for parameters derived from the OH and TMB tasks, and for the *year-to-year* study only for parameters from the OH task. For the TMB task in the *within-season* study, the largest practice effect was found for the Test Time parameter, a notable finding given its common clinical use [23,24]. Similar practice effects in the pencil and paper version of the TMB task have been reported previously [33]. Also of interest, the proportion of total parameters with δ values of 0.50 or greater from the first to second assessment were similar whether they were separated by one to two months (*within-season* study) or one year (*year-to-year* study). This finding suggests that the practice effects associated with completing a baseline assessment with these tasks does not necessarily diminish with time for most parameters. As with our prior work with the KINARM [12], performance plateaued between the second and third assessment. This performance plateau after the second administration of the KINARM tasks is consistent with changes in performance observed in athletes undergoing computerized and pencil and paper neurocognitive tests [33]. When baseline testing and re-assessing individuals post-injury, such practice effects could obscure the detection of impairments. Potential strategies to mitigate such an impact for the KINARM assessments include baseline testing individuals twice prior to the athletic season, or accounting for a known normative practice effect when evaluating post-injury performance.

We also studied Task Scores that measure global performance on each robotic assessment [25]. In the *within-season* study, reliability of Task Scores varied between tasks and time points, with only the VGR and PM Task Scores yielding moderate-to-good reliability across both sets of time points (S1 to S2, and S2 to S3). In contrast, for the *year-to-year* study, Tasks Scores had moderate-to-good reliability for all tasks except OHA across all assessments (Y1 to Y2, and Y2 to Y3). In both studies, practice effects on Task Scores were minimal. Derivation of a weighted Task Score, with greater emphasis on parameters with moderate-to-good test-retest reliability, may provide a means to further improve reliability of such composite scores [42].

As mentioned above, the reliability of the KINARM tasks utilized here was similar to other assessment tools currently being used to assess SRC-related deficits, such as ImPACT neurocognitive testing [9,33–35], SOT posturography [38], and symptom reporting [39]. Given that SRC is a diffuse injury with many potential overlapping pathologies, it is not always clear what to look for (e.g., many different signs/symptoms, cognitive impairment, sensorimotor deficits, balance impairment, vestibular features, oculomotor deficits, cervicogenic features, autonomic nervous system dysfunction). There may be subtle deficits, which if missed, may have significant consequences in a high-risk sporting context. There is also the challenge of athlete cover-ups as they often want to return to sport quickly. Recent evidence demonstrates that the window for physiological recovery may outlast symptom and clinical recovery [43,44]. SRC is a brain injury but the acute clinical symptoms largely reflect an impairment in neurological function rather than structural brain injury [44]. The KINARM potentially adds to previously developed SRC assessment approaches by examining additional elements of neurologic function from multiple systems and brain processes simultaneously (e.g., upper-extremity motor and sensory function) that could plausibly be impacted by SRC. A further benefit of the KINARM is that it can be custom programmed, and has the potential to be integrated with force plate and eye tracking technology. Thus, it is possible that new tasks designed to assess elements of neurologic function probed by ImPACT, SOT or oculomotor testing could be added to the repertoire of KINARM tasks used to assess SRC, or that more complex and demanding dual-tasks could be developed to measure subtle SRC-related deficits that are clinically significant when returning to high-risk sport participation. Lastly, the efficiency of the KINARM assessment (<20 minutes for all tasks used here) and its provision of immediate access to objective quantitative data and normative models for the KINARM Standard Tests suggests that it holds promise for overcoming many of the challenges associated with improving SRC assessment.

Although the test-retest reliability and practice effects appear similar across the *within-season* and *year-to-year* studies for most of the KINARM parameters, differences in the compositions of the study samples make it difficult to draw comparisons. For example, the *within-season* study included only male athletes from varsity team sports, while the *year-to-year* study included both male and female athletes from varsity team sports and national level individual sports. There is currently limited information available regarding sex differences in KINARM standard task performance. One study examined sex differences in performance of a version of a KINARM standard task [22]. Using the KINARM exoskeleton robot and a 9-target version of the PM task (KINARM endpoint robot and 4-target PM task used here), a significant sex difference was reported for the Absolute Error XY parameter, but no others [22]. Other work demonstrated sex differences in neuropsychological testing [45] and aspects of motor behaviour, such as visual reaction time [46]. Taken together, these studies suggest that sex could also potentially contribute to variability in KINARM task performance and reliability in our current study. The potential influence of type of athlete (i.e., sport) on KINARM task performance has not been studied previously, but it stands to reason that the varying skillsets required of athletes participating in different sports and at different levels of competition might be reflected in KINARM task performance. We are not currently powered to examine these potential effects further, but it is important to note that differences in sex and sport distribution could have plausibly impacted variability and reliability across the two studies reported here.

Another important consideration when interpreting the current work is that we included only healthy participants, which can lead to low inter-subject variability in performance and negatively impact measures of test-retest reliability. Consistent with this postulation, the ICCs for the VGR and PM tasks were slightly lower than what was found in individuals with acute stroke [15,17], a finding also reported in the test-retest reliability pediatric study that was conducted in healthy athletes [12]. Future work may consider evaluating the reliability of these tests in individuals with SRC, as test-retest reliability of these measures may be higher when determined in a sample with greater inter-subject variability. Additionally, we pooled participants with and without a history of concussion in our study samples. Although past work in pediatric athletes has indicated no effect of a distant history of concussion on performance of the KINARM tasks used here [13], only further work with larger, balanced groups can address whether performance or test-retest reliability is moderated by a self-reported history of physician diagnosed concussion and associated recovery time in young adult athletes. Lastly, it should also be noted that we cannot account for intrinsic variability in performance related to potential visual system deficits or individual changes that occurred between assessments. Visual acuity for participants is reported in the Results section; however, subtle problems that might be detected through examination by an optometrist or opthamologist (e.g., suboptimal refractive correction, slight ocular misalignment, subtle oculo-motor problems, etc.) could potentially impact performance on the majority of the KINARM tasks employed. Nevertheless, the within-subjects nature of the test-retest reliability study design should at least partly mitigate variability introduced by participants with potentially sub-clinical visual system problems.

Given the end goal of utilizing this technology for assessment of acute SRC and recovery, the feasibility of using the KINARM to this end should also be addressed. Prior work examined the feasibility of integrating the KINARM into an emergency department setting for neurologic assessment of mTBI [47]. The primary challenges faced in this emergency department study related to technological maintenance and the implementation of the large, relatively immobile KINARM device into a busy clinical environment [47]. These same challenges are certainly applicable to the integration of the KINARM device into a sport medicine clinic for assessment of SRC. We overcame technological challenges through continued collaboration with the support staff of the makers of the KINARM (BKin Technologies Ltd.) and involvement of researchers with skills in computer science. Rather than adapt a mobile KINARM, as done in prior work [47], we have a dedicated room in the sports medicine clinic for all KINARM testing, and a staff member whose primary tasks are to schedule, collect and compile the information obtained from the robotic assessments. Conducting truly acute assessments of SRC (i.e., sideline assessments or <24 hours) with this device is unlikely given its size (>800 lbs) and electrical requirements, but not necessarily impossible. Rather, the most realistic and clinically meaningful use of the KINARM for SRC may be in reliably, efficiently, and objectively tracking recovery relative to a previously conducted baseline test. Nevertheless, further work is needed to gain a better understanding of the full potential real-world applicability of robotic technology for SRC assessment.

## Conclusions

We found moderate-to-good test-retest reliability and minimal practice effects in healthy, young adult athletes for most of the KINARM task parameters evaluated. While differences in study sample composition precluded direct comparison between the *within-season* and *year-to-year* studies, the reliability and magnitude of practice effects appeared similar across the two timeframes of testing. Future work with the KINARM may consider focusing on parameters known to have high test-retest reliability, refining methods of deriving composite scores to increase their reliability, and development of additional tasks that are more demanding and further involve cognitive, postural and oculomotor function. Overall, our findings support continued study of the feasibility and effectiveness of the KINARM for prospectively assessing the effects of SRC on neurologic function.

## Supporting information

S1 File Data for test-retest reliability studies. (XLSX)
